# Conceptual Plurality in Transition Programmes for Newly Hired Nurses: An Umbrella Review

**DOI:** 10.3390/nursrep16050163

**Published:** 2026-05-13

**Authors:** Marcello Torre, Cristina Arrigoni, Rosario Caruso, Antonio Maria Giuseppe Staffa, Desiree Lucà, Arianna Magon

**Affiliations:** 1 Department of Biomedicine and Prevention, University of Rome Tor Vergata, 00133 Rome, Italy; marcello.torre@asst-settelaghi.it; 2 Direction of Healthcare Professions, Azienda Socio Sanitaria Territoriale Sette Laghi, 21100 Varese, Italy; antoniomariagiuseppe.staffa@asst-settelaghi.it (A.M.G.S.); desiree.luca@asst-settelaghi.it (D.L.); 3 Department of Public Health, Experimental and Forensic Medicine, Section of Hygiene, University of Pavia, 27100 Pavia, Italy; cristina.arrigoni@unipv.it (C.A.); arianna.magon@unipv.it (A.M.); 4 Department of Biomedical Sciences for Health, University of Milan, 20133 Milan, Italy; 5 Health Professions Research and Evidence Transfer Unit, IRCCS MultiMedica, 20099 Sesto San Giovanni, Italy

**Keywords:** transition-to-practice, new graduate nurses, nurse residency programmes, preceptorship, professional transition, umbrella review

## Abstract

**Background/Objectives**: Nurse transition programmes are widely implemented to support newly hired nurses and promote workforce retention. Despite the growing number of published reviews, conceptual inconsistency and methodological heterogeneity limit the interpretability and cumulative value of the evidence. This umbrella review aimed to synthesise and critically examine review-level evidence on nurse transition programmes, clarifying programme typologies, contexts, methodological approaches, reported outcomes, and thematic patterns. **Methods**: An umbrella review was conducted in accordance with PRISMA 2020 guidance. Systematic searches were performed in CINAHL, PubMed, Scopus, Web of Science, and Google Scholar, supplemented by citation tracking. **Results**: Fourteen reviews published between 2010 and 2025 were included: 12 reviews of primary studies and two reviews of secondary evidence (one umbrella review and one meta-review). Programme models and outcome measures were highly heterogeneous, and primary study overlap was slight (CCA = 2.55), indicating that reviews in the corpus drew on largely non-overlapping sets of primary studies. Transition programmes for new nurses commonly use one-on-one preceptorships with supernumerary practice, simulation-based learning, and active methods like case studies and reflective journaling to build competence and confidence. Their duration varies from a few days to 12 months, aligning with the progressive learning curve of new graduates. Professional outcomes, particularly competence and confidence, were consistently reported, whereas organisational outcomes, such as retention, showed mixed, methodologically constrained evidence. Patient-level outcomes were rarely examined. Thematic analysis revealed a shift over time from individual professional readiness towards implementation and organisational considerations. **Conclusions**: Given this conceptual plurality, there is an urgent need to standardise key indicators for evaluating the effectiveness of nurse transition programmes across healthcare settings globally.

## 1. Introduction

Healthcare systems globally face persistent nursing shortages that threaten service capacity, care continuity, and organisational sustainability [1,2,3]. Importantly, the challenge lies not only in increasing the number of available nurses but also in ensuring that healthcare organisations can effectively integrate and retain newly hired staff, particularly during the critical early employment period, when attrition risk is highest [4,5,6]. Workforce retention has therefore become a strategic organisational priority, increasing attention to interventions intended to support nurses’ entry into new roles and practice settings [7,8].

Research on nurse retention and turnover consistently points to a constellation of professional and organisational determinants, including job satisfaction, work engagement, leadership and supervisory support, professional development opportunities, organisational commitment, and the practice environment [9,10,11,12,13]. Interventions focused on the early employment phase have been identified as potentially modifiable organisational levers that may influence these determinants. Among such interventions, programmes designed to support nurses’ transition into professional roles have attracted sustained attention [14]. However, the literature describing these programmes is marked by substantial conceptual and terminological variability, which complicates both synthesis and translation into organisational practice [15,16].

Across the review literature, terms such as orientation, onboarding, transition-to-practice, nurse residency, preceptorship, mentorship, and hybrid formulations are frequently used without consistent differentiation [17,18,19,20]. In some sources, orientation denotes a short-term, task- and unit-focused process, whereas onboarding and transition are treated as a longer trajectory of professional development and organisational socialisation [21]. In other sources, these distinctions are not maintained, and identical labels are applied to programmes that differ meaningfully in content, duration, intensity, and delivery mechanisms [17].

This definitional uncertainty has practical implications because the outcomes attributed to these programmes extend beyond competence acquisition [22]. Contemporary workforce pressures, including changing expectations among early-career nurses and broader disruptions to working conditions in the post-pandemic period, have further foregrounded organisational interest in transition support, while expanding the set of outcomes considered relevant [23]. These outcomes span individual professional domains (e.g., confidence, readiness for practice, perceived competence), team and organisational domains (e.g., socialisation, support structures, workload), and workforce metrics (e.g., retention, turnover, and longer-term sustainability) [17,18,19,21,22,23].

Over the past decade, the growth of primary studies in this area has been paralleled by a proliferation of evidence syntheses [9,10,14,15,16,18,19]. Reviews of primary research have addressed specific programme families (notably nurse residency and transition-to-practice models) and, in some instances, have aggregated very large bodies of evidence, with individual reviews including more than 130 primary studies [24]. In parallel, reviews of reviews (umbrella reviews and meta-reviews) have emerged to provide higher-level summaries [25]. Yet the availability of multiple reviews has not translated into conceptual consolidation or cumulative clarity [24,25].

Existing syntheses, both primary and secondary, remain heterogeneous in scope, methods, and analytic emphasis, and they frequently rely on partially overlapping but weakly shared sets of primary studies, even when they ostensibly address similar interventions [17,18,19,21,22,23]. Thus far, the available reviews of reviews inherit this heterogeneity when they synthesise diverse secondary sources that themselves differ in programme definitions, inclusion criteria, and outcome operationalization [25]. As a result, the review-level evidence base exhibits limited internal coherence, making it difficult to determine which programme types are being discussed, which implementation determinants are consistently reported, and which professional and organisational outcomes are supported by convergent review-level evidence rather than isolated findings.

In this context, the presence of many reviews should not be interpreted as evidence of saturation [26,27]; rather, it signals fragmentation across concepts, programme typologies, and synthesis approaches. What is currently missing is a higher-order synthesis that explicitly addresses conceptual plurality, maps programme types and contexts across reviews, and situates reported implementation facilitators/barriers and outcomes within the characteristics of the reviews themselves (e.g., publication period, review type, and disciplinary orientation). An umbrella review is, therefore, methodologically warranted to consolidate and interrogate review-level evidence while making heterogeneity transparent rather than obscuring it. A single umbrella review on this topic is currently available [25]; however, it was restricted to retention and turnover outcomes following introduction programmes for newly graduated nurses in hospital settings. Review-level evidence across the broader spectrum of transition programmes, encompassing orientation, onboarding, transition-to-practice, residency, preceptorship, and mentorship, has not yet been consolidated at a higher-order synthesis level that explicitly interrogates conceptual plurality. Accordingly, the aim of this umbrella review is to synthesise and critically examine review-level evidence on nurse transition programmes for newly hired nurses. In addressing this aim, the review maps programme typologies and contexts, characterises the methodological approaches of existing reviews, identifies implementation facilitators and barriers, summarises professional and organisational outcomes, and examines thematic patterns in relation to review characteristics.

## 2. Materials and Methods

### 2.1. Design

This study was conducted as an umbrella review following established methodological guidance [26,27]. The design was selected to address a literature base characterised by a high volume of secondary syntheses, substantial conceptual heterogeneity, and limited overlap among the primary studies included across reviews. In contrast to reviews that privilege a single synthesis approach, this umbrella review intentionally encompassed all major forms of evidence synthesis relevant to the topic, including systematic reviews, integrative reviews, scoping reviews, and reviews of reviews (umbrella and meta-reviews). The protocol for this study was developed a priori and registered in the PROSPERO database (registration number: CRD42021227502). Any deviations from the original protocol are described and justified in the present manuscript.

In this review, conceptual plurality is understood not merely as variation in terminology, but as the coexistence of partially overlapping yet non-equivalent programme concepts that differ in scope, duration, level of formalisation, core components, and organisational embedding. For this reason, an inclusive umbrella review design was adopted because nurse transition programmes, commonly labelled as orientation, onboarding, transition-to-practice, residency, preceptorship, mentorship, or hybrid models, have been examined across reviews that differ markedly in purpose, methodological orientation, and analytical focus [8,9,10,11,12,13,14,15,16,19,20]. This design enables consolidation of secondary evidence while preserving transparency regarding conceptual plurality and methodological diversity, consistent with the research questions detailed below.

The protocol for this umbrella review was developed a priori and published elsewhere, ensuring methodological transparency and reducing the risk of selective reporting [28]. The conduct and reporting of the review were informed by established best-practice guidance for umbrella reviews and evidence synthesis, including principles outlined by the Joanna Briggs Institute and relevant reporting standards for reviews of reviews, such as by adhering to The Preferred Reporting Items for Systematic reviews and Meta-Analyses (PRISMA) statement 2020 [26,29].

### 2.2. Research Questions

To operationalise the review aim, the following research questions were developed: How have nurse transition programmes—including orientation, onboarding, residency, and related organisational models—been conceptualised, synthesised, and evaluated across the existing review-level literature? To operationalize the overarching question, the following specific research questions were developed: (RQ1) What types of transition programmes for newly hired nurses are described across existing reviews? (RQ2) In which geographical and clinical contexts have transition programmes been examined in the review literature? (RQ3) How have existing reviews synthesised evidence on transition programmes, and which methodological approaches have been adopted? (RQ4) What facilitators and barriers to the implementation of transition programmes are reported across reviews? (RQ5) What professional and organisational outcomes are associated with transition programmes for newly hired nurses according to the review-level evidence? (RQ6) How do thematic patterns emerging from existing reviews relate to the characteristics of those reviews?

### 2.3. Eligibility: Inclusion and Exclusion Criteria

Criteria were structured to capture the full breadth of secondary evidence addressing nurse transition programmes, while excluding sources that would not contribute to review-level synthesis or conceptual clarification.

#### 2.3.1. Types of Evidence Sources

Eligible sources were reviews of research, including systematic reviews, integrative reviews, scoping reviews, and reviews of reviews (umbrella or meta-reviews). These review types were included regardless of whether they employed quantitative, qualitative, or mixed-methods synthesis approaches. The deliberate inclusion of reviews (umbrella and meta-reviews) as eligible sources represents a non-standard but methodologically motivated choice. Conventional umbrella reviews, as defined in canonical methodological guidance [26,27], are designed as systematic reviews of systematic reviews and typically exclude higher-order syntheses. In the present study, however, the unit of analysis is the published review itself, and the phenomenon under investigation is the conceptual plurality and structural heterogeneity of the review-level evidence based on nurse transition programmes. Because the recent proliferation of umbrella and meta-reviews constitutes a defining feature of this evidence landscape, and therefore part of the phenomenon of interest, systematically excluding them would have led to an incomplete and biassed representation of how the field has been synthesised. The broader literature on overviews of reviews acknowledges that eligibility frameworks may be adapted when the synthesis aim is descriptive, mapping, or conceptual rather than effect-estimative [30,31]. We therefore adopted an inclusive design while implementing specific analytic safeguards (see Section 2.8 and Section 3.1) to make the risk of primary-study overlap transparent and quantifiable. Reviews were eligible provided that they reported a systematic or explicitly described search strategy and synthesised findings from multiple primary studies.

Primary studies, narrative commentaries, editorials, opinion papers, protocols without accompanying results, and methodological papers that did not report empirical synthesis were excluded. Reviews focusing exclusively on educational curricula without reference to organisational entry or transition into professional roles were also excluded.

#### 2.3.2. Population

Reviews were eligible if they focused on nurses entering new professional roles or organisational contexts, including newly graduated, newly qualified, newly hired, or early-career nurses. Reviews addressing mixed healthcare professional populations were included only when nurse-specific findings were reported separately or constituted a substantial focus of the synthesis. Reviews focusing exclusively on students without reference to post-employment transition were excluded.

#### 2.3.3. Phenomenon of Interest/Interventions

The phenomenon of interest comprised organisational-level nurse transition programmes that support nurses during entry into professional practice or new employment contexts. Eligible reviews examined one or more programmes commonly labelled as orientation, onboarding, transition-to-practice, nurse residency, preceptorship, mentorship, or hybrid models combining multiple components. Reviews were included irrespective of programme duration, intensity, or level of formalisation.

Given the study’s focus on conceptual plurality, eligibility was determined by the programme’s functional intent (i.e., structured support for professional and organisational transition) rather than by strict terminological definitions. Reviews that explicitly addressed organisational integration, professional socialisation, or supported transition during early employment were therefore eligible even when programme labels varied.

#### 2.3.4. Outcomes

Reviews were eligible if they reported professional, organisational, or implementation-related outcomes associated with nurse transition programmes. These outcomes included, but were not limited to, professional competence, confidence, readiness for practice, job satisfaction, engagement, wellbeing, retention, turnover, organisational commitment, and workforce stability, as well as facilitators and barriers to programme implementation. Reviews were not required to report quantitative effect estimates to be eligible.

#### 2.3.5. Context

Reviews conducted in any healthcare setting (e.g., acute care, community, long-term care, critical care) and any geographical context were eligible. No restrictions were applied based on country income level or healthcare system characteristics, in line with the objective of mapping the global review-level evidence base.

#### 2.3.6. Language and Time Frame

Only reviews published in English were included. No restrictions were applied on the year of publication, allowing examination of temporal patterns in review characteristics and thematic emphases.

### 2.4. Information Sources and Search Strategy

To identify relevant reviews, a comprehensive literature search was conducted across four major electronic databases with broad coverage of nursing, biomedical, and interdisciplinary research: the Cumulative Index to Nursing and Allied Health Literature (CINAHL), PubMed, Scopus, and Web of Science (WoS). These databases were selected to ensure the retrieval of reviews published in nursing-specific, clinical, and health services research journals.

In addition to electronic database searches, hand searching of the reference lists of all included reviews was undertaken, as well as Google Scholar (including grey literature). Backward and forward citation tracking was also performed to identify potentially relevant reviews not retrieved through database searching. Search dates and the number of records retrieved from each database were documented in detail. Where necessary, the corresponding authors of included reviews were contacted to clarify review methods or the scope of included interventions.

#### Search Strategy Development

The conceptual scope of the search was informed by the “population, intervention comparison, outcome” (PICO) framework [32], specifying the population as newly hired nurses, the intervention as onboarding-related transition programmes, comparators as any or none, and outcomes as any professional or organisational outcomes. However, because the unit of analysis of this study was published reviews rather than individual primary studies, inclusion and exclusion criteria were applied at the review level, and the search strategy was designed to be inclusive and sensitive rather than outcome-restrictive.

Given the exploratory nature of the review domain and the aim of synthesising heterogeneous secondary evidence, the search strategy was intentionally broad. This approach was adopted to maximise the retrieval of potentially relevant reviews across diverse terminologies and programme labels. Refinement and exclusion were subsequently applied during the screening phase based on predefined eligibility criteria rather than during the initial search. This strategy is consistent with best-practice guidance for umbrella reviews in conceptually fragmented fields. Table 1 presents the key conceptual elements underpinning the search strategy.

Search terms were developed to capture variation in programme labels and terminology commonly used to describe nurse transition interventions, including orientation, onboarding, transition-to-practice programmes, mentorship, and preceptorship. Population-related terms focused on nurses and their newly hired or newly graduated status. No a priori restrictions were applied to outcomes or comparators, allowing comprehensive mapping of professional, organisational, and implementation-related outcomes reported in the review literature. Boolean operators and database-specific subject headings were used as appropriate, following established guidance for systematic literature searching.

Although the search strategy was informed by population and intervention concepts, eligibility decisions were applied exclusively at the level of published reviews, in line with the study’s review-level focus. Table 2 reports the final database-specific search strings, the number of records retrieved, and the dates of database consultation.

### 2.5. Selection Process

Study selection was conducted in accordance with PRISMA 2020 guidance using a two-stage screening process, comprising (i) title and abstract screening and (ii) full-text assessment [29]. All records retrieved from the electronic database searches were exported into reference management software, and duplicate records were identified and removed prior to screening.

At the first stage, titles and abstracts were independently screened by two reviewers to assess potential eligibility against the predefined inclusion and exclusion criteria. This initial screening was intentionally inclusive, with records retained for full-text review whenever eligibility could not be clearly determined from the title or abstract alone. Records judged by both reviewers to be clearly irrelevant were excluded at this stage.

At the second stage, full texts of all potentially eligible reviews were retrieved and independently assessed for inclusion by the same two reviewers. Full-text screening focused on review-level eligibility, including confirmation of review type, population, intervention scope, and relevance to nurse transition programmes. Reasons for exclusion at the full-text stage were documented in detail to ensure transparency and reproducibility.

Disagreements at either screening stage were resolved through discussion and consensus. When consensus could not be reached, a third reviewer was consulted to adjudicate. Inter-reviewer agreement was not used as an exclusion criterion but was monitored throughout the screening process to ensure consistent application of eligibility criteria.

### 2.6. Data Extraction Process

Data extraction was conducted using a standardised, purpose-built extraction form developed a priori to capture review-level characteristics and findings relevant to the objectives of the umbrella review. The extraction form was designed to reflect the analytical focus of the study, with particular attention to conceptual plurality, methodological heterogeneity, and variation in reported programme components and outcomes.

Before full data extraction, the extraction form was pilot-tested on a subset of the included reviews (approximately 10–15% of the final sample). Pilot testing aimed to assess clarity, completeness, and consistency of the extraction items, and to ensure that the form adequately captured variations in terminology, programme typologies, and synthesis approaches across reviews. Based on this pilot phase, minor refinements were made to improve item definitions and reduce ambiguity, without altering the underlying analytical framework.

Following pilot testing, data extraction was performed independently by two reviewers for all included reviews. Extraction focused exclusively on review-level data, rather than on re-extracting findings from individual primary studies. Extracted information included bibliographic details, review type and methodological characteristics, population and setting, programme labels and typologies, reported facilitators and barriers to implementation, professional and organisational outcomes, and key conceptual or thematic emphases described by the review authors.

To ensure consistency across heterogeneous review designs and reporting styles, reviewers were instructed to extract data as reported by the original review authors, avoiding reinterpretation or reclassification during extraction. Where information was unclear, inconsistently reported, or missing, this was recorded as “not reported” rather than inferred. Any discrepancies between reviewers during the extraction process were resolved through discussion and consensus. When agreement could not be reached, a third reviewer was consulted to adjudicate.

The final extracted dataset constituted the basis for subsequent descriptive, comparative, and thematic synthesis. This structured, duplicate-extraction process was implemented to enhance transparency, reduce extraction bias, and ensure reproducibility, while remaining aligned with the umbrella review’s unit of analysis.

#### Data Item

Data items were defined a priori to ensure systematic, transparent, and reproducible extraction of information relevant to the objectives of this umbrella review. Data extraction was conducted exclusively at the review level, consistent with the study’s unit of analysis, and was designed to capture descriptive characteristics of the included reviews, as well as conceptual, methodological, and thematic elements necessary to examine heterogeneity across the review literature.

For each included review, bibliographic and descriptive characteristics were extracted, including first author, year of publication, journal and disciplinary focus, geographical scope, review type, stated aims, time period covered, number of primary studies included, and the nature of the evidence base synthesised (primary studies or secondary reviews). Information regarding the populations addressed by the reviews was also collected, with particular attention to the target groups described by the authors, such as newly graduated, newly qualified, or newly hired nurses. Where reported, data on clinical and organisational settings and the geographical contexts of the primary studies synthesised within each review were extracted.

To address conceptual plurality, data extraction captured the terminology used by review authors to describe nurse transition programmes, including labels such as orientation, onboarding, transition-to-practice, residency, preceptorship, mentorship, and hybrid models. Descriptions of programme scope, duration, and level of formalisation were extracted when available, along with any explicit definitions or conceptual frameworks used to characterise these programmes. This information was essential for comparing programme typologies and examining variation in how similar interventions were conceptualised across reviews.

Data were also extracted on implementation-related determinants reported at the review level, including facilitators and barriers to programme delivery and sustainability, as well as contextual or organisational factors influencing implementation where described. In addition, outcomes associated with nurse transition programmes were recorded, encompassing professional outcomes (such as competence, confidence, readiness for practice, wellbeing, and job satisfaction), organisational outcomes (including retention, turnover, organisational commitment, and workforce stability), and, where reported, economic or system-level outcomes.

Methodological and analytical features of each review were documented, including the synthesis methods employed, the use of critical appraisal, and limitations and implications identified by the review authors. Finally, key themes or conceptual categories reported in each review were extracted, along with information on thematic emphases and any reported relationships between themes and review characteristics, such as publication year, review type, or disciplinary orientation.

All data items were extracted as reported by the original review authors. When information was missing, unclear, or inconsistently reported, this was documented as not reported rather than inferred. No reanalysis or reinterpretation of primary study data was undertaken beyond the level of synthesis presented in the included reviews. The extracted data formed the basis for the descriptive, comparative, and thematic syntheses presented in the Results section, including tabular summaries and visual mapping of thematic patterns across reviews.

### 2.7. Critical Appraisal of Included Reviews

The methodological quality of included reviews was appraised to characterise the overall robustness of the review-level evidence base and to support transparent interpretation of findings. Given the inclusion of heterogeneous review types, including systematic reviews, integrative reviews, scoping reviews, and reviews of reviews, critical appraisal was conducted using tools appropriate for reviews of evidence rather than instruments designed for primary studies.

The Joanna Briggs Institute (JBI) Critical Appraisal Checklist for Systematic Reviews and Research Syntheses was used as the primary appraisal framework [33]. This tool was selected because it is widely recommended for umbrella reviews and allows assessment of key methodological domains relevant across different types of reviews, including clarity of review questions, appropriateness of inclusion criteria, adequacy of search strategies, transparency of study selection and data extraction processes, assessment of methodological quality of included studies, appropriateness of synthesis methods, and consideration of publication bias.

Critical appraisal was conducted independently by two reviewers for all included reviews. Discrepancies in appraisal judgments were resolved through discussion and consensus, with involvement of a third reviewer when necessary. Appraisal focused on the methodological conduct and reporting of each review, rather than on the quality of individual primary studies included within those reviews.

Given the objectives of this umbrella review, critical appraisal results were used descriptively rather than as a basis for excluding reviews or weighting findings. This decision reflects the purpose of the study, which was to examine conceptual, methodological, and thematic patterns across the review literature rather than to produce pooled estimates of effectiveness. Excluding reviews on the basis of quality alone would have risked further narrowing an already fragmented evidence base and obscuring how methodological variability contributes to conceptual plurality in the field.

### 2.8. Assessment of Overlap Across Reviews

To assess the degree of overlap of primary studies across included reviews, the Corrected Covered Area (CCA) was calculated [34]. CCA is a quantitative measure specifically developed for umbrella reviews to estimate the extent to which primary studies are shared across multiple reviews. It accounts for the number of unique primary studies, the total number of included reviews, and the frequency with which primary studies are repeated across reviews. A citation matrix was constructed to map the inclusion of primary studies across reviews. The CCA was calculated using the following formula:

(1) $$\mathrm{CCA}\;=\;\frac{N-r}{\left(r\times c\right)-r}$$

where *N* represents the total number of primary study occurrences across all reviews (i.e., the sum of included primary studies counting duplicates), *r* represents the number of unique primary studies, and *c* represents the number of included reviews.

The CCA was calculated using established formulas and interpreted according to published thresholds, with values below 5% indicating slight overlap, 6–10% moderate overlap, 11–15% high overlap, and values above 15% indicating very high overlap. The CCA was used descriptively to characterise the degree of overlap within the evidence base and to contextualise findings, rather than to exclude reviews or weight results.

Because the inclusion of reviews is unusual in umbrella review methodology and introduces a theoretical risk of double-counting of primary evidence, an additional sensitivity analysis was pre-specified. For this cross-check, the primary studies indexed within the two included umbrella/meta-reviews [25,35] were traced back through their constituent reviews and incorporated into an expanded citation matrix covering all 14 reviews. A second CCA was then computed over this expanded pool to assess the robustness of the overlap estimate.

## 3. Results

### 3.1. Study Selection

**Figure 1:**
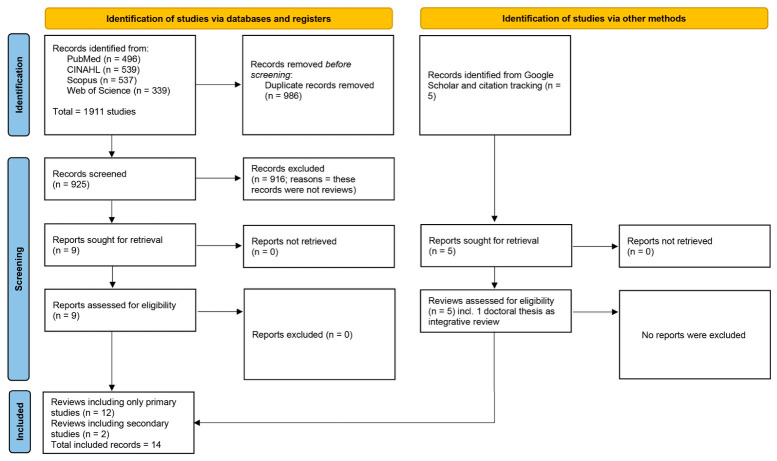
PRISMA 2020 flow diagram.

The database search identified a total of 1911 records, including 496 from PubMed, 539 from CINAHL, 537 from Scopus, and 339 from Web of Science. After the removal of 986 duplicate records, 925 records remained for title and abstract screening. Of these, 916 records were excluded at the screening stage, primarily because they were not review articles and thus did not meet the review-level eligibility criteria (see Figure 1).

Nine reports were subsequently sought for full-text retrieval, and all were successfully retrieved and assessed for eligibility. All nine reports met the inclusion criteria and were retained. In addition to database searching, five records were identified through supplementary sources (Google Scholar and backward/forward citation tracking), including one doctoral thesis classified as an integrative review. All five records were retrieved, assessed for eligibility, and included. Overall, 14 reviews were included in the umbrella review [24,25,35,36,37,38,39,40,41,42,43,44,45,46]. Of these, 12 synthesised only primary studies [24,36,37,38,39,40,41,42,43,44,45,46], while two were reviews of secondary evidence: one umbrella review [25] and one meta-review with integrative elements [35]. The study selection process is illustrated in the PRISMA 2020 flow diagram (Figure 1).

The 12 reviews synthesising only primary studies [24,33,34,35,36,37,38,40,41,42,43,44] contributed 239 primary study occurrences, corresponding to 194 unique primary studies, and yielded a CCA of 2.55% (slight overlap, indicating that reviews in the corpus drew on largely non-overlapping sets of primary studies). In interpretive terms, this means that across the 12 reviews of primary studies, the same primary study was rarely included in more than one review: approximately 97.5% of the primary study occurrences corresponded to unique studies.

The included reviews, therefore, drew on largely independent bodies of primary evidence rather than on a shared core of frequently re-synthesised studies. The sensitivity CCA, computed over the expanded pool of 14 reviews after incorporating the primary studies transitively contributed by the two umbrella/meta-reviews [25,35], did not exceed 3.0%, remaining within the slight-overlap range. This finding indicates that the inclusion of secondary-evidence reviews did not materially alter the overlap profile of the review-level evidence base.

### 3.2. Methodological Quality of Included Reviews

**Figure 2:**
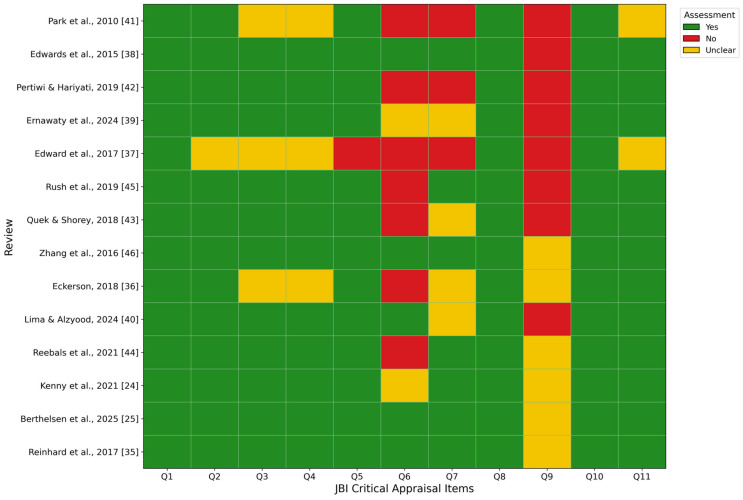
JBI Critical Appraisal Checklist for Systematic Reviews and Research Syntheses. Note: Q1: Is the review question clearly and explicitly stated? Q2: Were the inclusion criteria appropriate for the review question? Q3: Was the search strategy appropriate? Q4: Were the sources and resources used to search for studies adequate? Q5: Were the criteria for appraising studies appropriate? Q6: Was critical appraisal conducted by two or more reviewers independently? Q7: Were there methods to minimise errors in data extraction? Q8: Were the methods used to combine studies appropriate? Q9: Was the likelihood of publication bias assessed? Q10: Were recommendations for policy and/or practice supported by the reported data? Q11: Were the specific directives for new research appropriate? [24,25,35,36,37,38,39,40,41,42,43,44,45,46].

Figure 2 presents the methodological quality of the included reviews as assessed using the Joanna Briggs Institute (JBI) Critical Appraisal Checklist for Systematic Reviews and Research Syntheses. Overall, the methodological quality of the reviews was variable, reflecting substantial heterogeneity in review design, rigour, and reporting practices.

Most reviews adequately addressed core methodological domains related to the clarity of the review question, the appropriateness of inclusion criteria, and the description of search strategies (Items Q1–Q4), which were generally rated as “Yes” across the majority of included reviews. This indicates that the foundational elements of review conduct were consistently reported. Similarly, most reviews clearly described the characteristics of included studies and the methods used to synthesise findings (Items Q8, Q10, and Q11).

In contrast, several critical appraisal domains related to the assessment and management of bias were less consistently addressed. Items concerning the appraisal of methodological quality of included studies, the consideration of risk of bias in interpretation of results, and the assessment of publication bias (Items Q6–Q9) showed a higher frequency of “No” or “Unclear” ratings. This pattern was particularly evident among integrative reviews and earlier systematic reviews, in which critical appraisal procedures were either partially reported or not explicitly described.

### 3.3. Characteristics of Transition Programme Types Described Across Reviews

Fourteen reviews were included [24,25,35,36,37,38,39,40,41,42,43,44,45,46], comprising 12 reviews of primary studies and two reviews of secondary evidence (one umbrella review [25] and one meta-review [35]). Reviews were published between 2010 and 2025 and synthesised literature spanning from 1980 to 2025. The methodological characteristics of the included reviews are summarised in Table 3. Across the 14 included reviews, substantial variation was observed in review type, the study designs synthesised, the approaches to critical appraisal, and the methods of synthesis. Regarding synthesis approaches, narrative synthesis predominated across review types. A smaller number of reviews employed thematic synthesis, particularly when synthesising qualitative evidence. Mixed-methods systematic reviews combined narrative and thematic approaches, while umbrella and meta-reviews adopted review-of-reviews synthesis methods.

As shown in Table 4, considerable heterogeneity was observed across reviews in terms of programme terminology, populations, settings, and outcomes. Most included reviews adopted integrative or systematic review designs, alongside one scoping review and two reviews of reviews (one umbrella review and one meta-review). Reviews of primary research varied widely in scope, ranging from 9 to 130 primary studies, whereas umbrella and meta-reviews synthesised evidence from previously published systematic reviews. Across reviews, primary study overlap was limited, indicating fragmentation of the underlying evidence base.

A wide range of programme labels was used, including orientation, transition-to-practice, nurse residency, preceptorship, mentorship, onboarding, and hybrid models combining multiple components. Orientation programmes were most frequently examined, particularly in earlier reviews, while transition-to-practice and residency programmes were commonly addressed in hospital and acute care settings. Preceptorship- and mentorship-based models were examined either as standalone interventions or as elements of broader transition programmes. Explicitly labelled onboarding programmes were primarily examined in recent reviews and umbrella-level syntheses.

Most reviews focused on newly graduated or newly qualified nurses, with fewer explicitly addressing newly hired nurses regardless of prior experience. Healthcare settings were predominantly hospital-based, although several reviews included multiple settings or focused on specific contexts such as critical care.

Professional outcomes, including competence, confidence, readiness for practice, and stress, were most consistently reported across reviews. Organisational outcomes, particularly retention and turnover, were also frequently examined, especially in reviews of residency and transition-to-practice programmes. Reviews of reviews tended to emphasise broader organisational outcomes, such as engagement and workforce stability.

### 3.4. Programme Models and Defining Features of Nurse Transition Interventions Across Reviews

Table 5 illustrates marked heterogeneity in how nurse transition interventions are structured and operationalised across the review literature, with programme models differing not only in content and duration but also in their degree of formalisation and intended scope. Across the included reviews, six main programme models were identified, differing in core components, duration, level of formalisation, and target populations.

**Figure 3:**
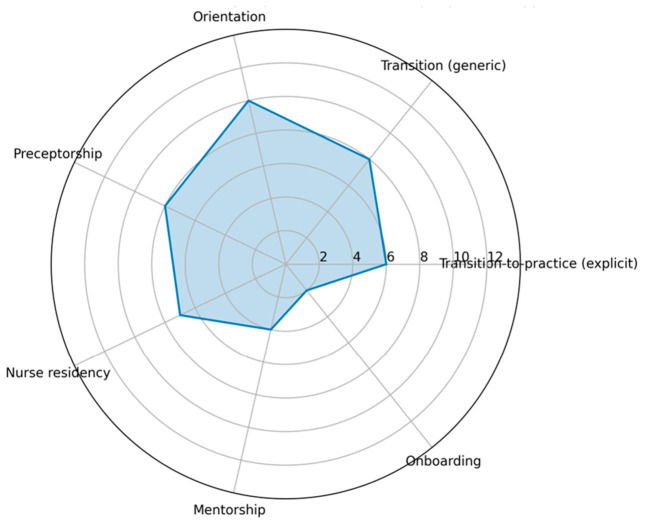
Radar plot of transition programme models reported in the included reviews. Note: The figure provides a more granular representation of terminology used in the review literature. In particular, the broader transition programme domain is visually split into “Transition (generic)” and “Transition-to-practice (explicit)”, whereas these are grouped within a higher-order category in Table 5.

Figure 3 provides a visual summary of the relative frequency with which different nurse transition programme models are reported across the included reviews, highlighting the predominance of orientation, transition-to-practice, and preceptorship-based approaches, and the more limited explicit use of the term “onboarding” in the review literature.

Orientation programmes were the most consistently reported model and were typically characterised by classroom-based education, unit-level orientation, supervised practice, and feedback mechanisms [38,39,41,42]. These programmes were generally short- to medium-term in duration and exhibited considerable variability in their level of formalisation, most commonly targeting newly graduated nurses.

Transition-to-practice programmes were described as more structured, longitudinal interventions that integrate formal education, supervised clinical practice, competency development, and professional socialisation [24,44,45]. These programmes were typically medium- to extended-duration and ranged from semi-formal to formal models, predominantly addressing the needs of newly graduated nurses.

Nurse residency programmes represented the most formalised model across reviews [25,35,36,45]. They were consistently described as extended interventions, often lasting 6 to 12 months, that combined classroom education with prolonged supervised practice, competency-based progression, and formal evaluation processes. Residency programmes primarily targeted newly hired nurses, most often newly graduated nurses, and were reported in both primary and secondary reviews.

Preceptorship-based models focused on one-to-one clinical supervision, experiential learning, and feedback, with substantial variability in duration and formalisation [37,40,43]. These models were most frequently reported in relation to newly graduated nurses, although some reviews included mixed populations. Mentorship-based models, in contrast, emphasised relational and psychosocial support alongside professional development and were generally described as semi-formal, with variable duration.

Hybrid models were identified in two reviews that combined elements of orientation, residency, transition-to-practice, mentorship, or preceptorship [24,38]. These models exhibited high variability in structure and formalisation but reflected attempts to integrate multiple components within a single programme framework.

### 3.5. Facilitators and Barriers to the Implementation of Transition Programmes Across the Included Reviews

Table 6 illustrates that facilitators and barriers to transition programme implementation operate across multiple levels and interact dynamically. Reviews consistently indicated that the effectiveness of transition programmes cannot be attributed to isolated components but depends on the alignment of individual readiness, interpersonal support, organisational structures, and system-level conditions. Facilitators and barriers to the implementation of transition programmes were consistently reported across the included reviews and could be organised analytically into four interrelated levels: individual, team or interpersonal, organisational, and system or structural.

### 3.6. Professional, Organisational, and Patient-Level Outcomes Reported Across Reviews

The review-level evidence suggests that nurse transition programmes are most consistently associated with improvements in professional outcomes and, to a lesser extent, organisational retention-related outcomes. In contrast, evidence regarding patient-level effects and system-wide organisational impacts remains sparse, fragmented, and methodologically underdeveloped.

Across the included reviews, outcomes associated with nurse transition programmes were predominantly reported at the professional and organisational levels, while patient-level outcomes were infrequently examined. As summarised in Table 7, the strongest and most consistent evidence concerned professional outcomes related to competence, confidence, and readiness for practice. Most reviews reported improvements in newly hired nurses’ clinical competence and self-confidence following participation in orientation, transition-to-practice, residency, or preceptorship-based programmes, although the magnitude and measurement of these effects varied considerably across studies and reviews.

Professional socialisation outcomes, including sense of belonging, professional identity development, and job satisfaction, were also frequently reported, particularly in reviews examining transition-to-practice, preceptorship, and mentorship models. However, findings in this domain were more heterogeneous, with some reviews reporting positive associations and others describing mixed or context-dependent effects. Outcomes related to stress reduction and the subjective transition experience were reported less consistently and showed mixed directions of association, reflecting variability in programme intensity, organisational context, and outcome measurement.

Organisational outcomes were most commonly examined in relation to retention, turnover, and intention to stay. Reviews focusing on nurse residency and structured transition programmes generally reported positive associations with retention and reduced turnover, although these outcomes were often inconsistently defined and measured across primary studies. Evidence for broader service-level or workforce outcomes, such as staffing stability and organisational performance, was limited and largely indirect, with few reviews providing robust or comparative data in this area.

Patient-level or clinical outcomes were rarely reported across the review literature. When mentioned, they were typically addressed indirectly through proxy indicators or inferred from improvements in nurse competence and workforce stability, rather than being explicitly measured. As a result, evidence linking transition programmes to patient outcomes remains inconclusive at the review level.

### 3.7. Evolution of Thematic Emphases in Nurse Transition Programme Reviews

**Figure 4:**
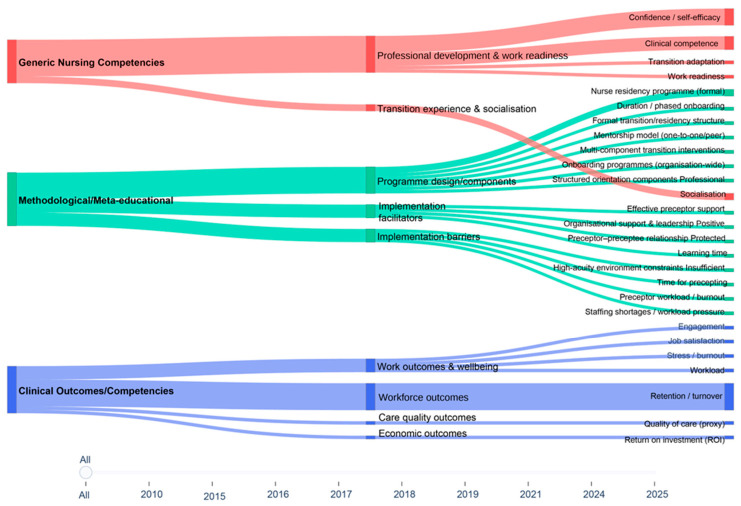
Alluvial plot illustrating thematic patterns across included reviews. Note: Flows represent the co-occurrence of macro-level thematic categories (L1), thematic domains (L2), and specific themes (L3) reported across reviews. Node width reflects the frequency with which themes appear in the review literature.

The analysis of thematic patterns across the included reviews indicates a structured and non-random relationship between dominant themes and key review characteristics, most notably publication period and review type, as visually synthesised in Figure 4. 

The alluvial representation reveals that thematic emphases vary systematically over time and across methodological approaches, reflecting shifts in how transition programmes for newly hired nurses have been conceptualised and synthesised within the review literature. Reviews published in the earlier phase of the evidence base, particularly those appearing up to 2015, are predominantly characterised by a thematic concentration on individual-level professional domains. In this period, the most salient thematic flows converge on constructs related to clinical competence, confidence, self-efficacy, and perceived work readiness. These reviews, largely integrative and systematic in nature, frame transition primarily as an educational and developmental intervention to support the transition of newly graduated or newly qualified nurses into clinical practice. Within this framing, the success of transition programmes is primarily interpreted through changes in individual professional capabilities and subjective readiness for practice, with limited attention to organisational or structural considerations.

In reviews published between 2016 and 2020, the thematic structure becomes more heterogeneous. While individual competence-related themes remain prominent, the alluvial plot demonstrates the emergence of additional thematic pathways related to the design and delivery of transition programmes. These include greater attention to formalised transition-to-practice models, preceptorship and mentorship structures, and the organisation of residency-type programmes. This intermediate phase reflects a gradual broadening of analytical focus, whereby transition is no longer viewed solely as an individual developmental process but increasingly as a structured intervention embedded within clinical and organisational contexts.

A further thematic shift is evident in the most recent reviews, particularly those published from 2021 onwards. In this phase, which includes a higher proportion of mixed-methods systematic reviews and umbrella or meta-reviews, the dominant thematic flows increasingly align with organisational and implementation-oriented domains. Themes related to workforce outcomes, such as retention and turnover, become more central, alongside attention to organisational facilitators and barriers, staff wellbeing, engagement, and, in a limited number of cases, economic considerations such as return on investment. The prominence of these themes suggests that more recent review-level syntheses conceptualise transition programmes as strategic organisational mechanisms with implications for workforce stability and system sustainability, rather than solely as interventions targeting individual nurse preparedness.

Thematic distributions also vary consistently according to review type. Integrative reviews display the greatest thematic breadth and occupy a connective position within the alluvial structure, linking individual-level competence themes with both implementation processes and organisational outcomes. This pattern reflects the methodological inclusiveness of integrative designs and their capacity to synthesise heterogeneous forms of evidence. In contrast, systematic reviews tend to exhibit more focused thematic trajectories, most commonly centred on predefined professional or workforce outcomes, consistent with their narrower scope and outcome-oriented synthesis strategies. Umbrella and meta-reviews demonstrate the strongest alignment with organisational- and system-level themes, reflecting both their secondary nature and their emphasis on higher-level synthesis across existing reviews.

Although the majority of included reviews are published within nursing journals, the thematic structure suggests an implicit evolution in disciplinary orientation over time. Earlier reviews predominantly reflect a nursing education and professional socialisation perspective, whereas more recent reviews increasingly adopt a workforce and organisational lens, emphasising the feasibility of implementation, sustainability, and organisational impact. Notably, patient-level or clinical outcomes appear only marginally within the thematic structure, with weak and infrequent flows, indicating that such outcomes remain peripheral within the review-level evidence on transition programmes for newly hired nurses.

Taken together, the alluvial analysis demonstrates that thematic patterns in the review literature are closely associated with both temporal and methodological characteristics of the reviews. The observed shift from an emphasis on individual professional readiness towards a focus on implementation processes and organisational outcomes reflects a broader evolution in the priorities of review-level synthesis, rather than evidence of a linear progression in programme effectiveness. This evolution underscores changing analytical perspectives within the field, as transition is increasingly framed as a system-level workforce intervention rather than solely as an individual professional development mechanism.

## 4. Discussion

The findings of this umbrella review highlight several methodological implications for understanding the structure and limitations of the existing review-level evidence on nurse transition programmes [24,25,35,36,37,38,39,40,41,42,43,44,45,46]. Most notably, the pronounced conceptual plurality identified across reviews is not merely a semantic issue but also reflects deeper methodological fragmentation that constrains synthesis, comparability, and the development of cumulative knowledge.

Although a conceptual distinction could be made between specific interventions (e.g., preceptorship and mentorship), the plurality of terms mainly relates to programme-level definitions. Thus, various programmes’ terms, such as orientation, onboarding, transition, and introduction, are often used interchangeably without a clear conceptual distinction, referring to a process that encompasses a range of formal and informal interventions that may substantially overlap across different programme-level labels [47]. This lack of conceptual standardisation undermines the internal coherence of individual reviews and complicates cross-review comparison. As a consequence, thematic convergence observed in the alluvial analysis often reflects shared terminology rather than shared intervention characteristics. Methodologically, this weakens the inferential value of review-level conclusions, as similarly named programmes may differ markedly in duration, intensity, formalisation, and organisational embedding.

The diversity of review types included, systematic, integrative, scoping, mixed-methods, and umbrella reviews, introduces additional layers of heterogeneity. While this plurality was intentionally embraced in the present umbrella review to reflect the full scope of secondary evidence, it also exposes fundamental differences in epistemological orientation, inclusion criteria, and synthesis strategies across reviews [30]. Integrative reviews tend to prioritise breadth and inclusivity, often synthesising heterogeneous study designs with limited critical appraisal, whereas systematic reviews typically adopt narrower eligibility criteria and outcome-focused syntheses. Reviews of reviews further abstract this evidence, amplifying higher-level themes while distancing findings from primary empirical contexts. These methodological differences shape not only what outcomes are reported, but also how effectiveness, implementation, and impact are conceptualised.

Moreover, the limited overlap of primary studies across reviews represents a critical structural constraint on the cumulative strength of the evidence base. The low CCA observed in this umbrella review indicates that most reviews draw on largely distinct sets of primary studies, even when addressing closely related programme types or research questions [48]. This pattern suggests that the proliferation of reviews has not led to progressive accumulation or triangulation of evidence, but rather to parallel syntheses of partially disconnected literature. From a methodological standpoint, this limits the degree to which review-level agreement can be interpreted as confirmation of robust effects, as convergence across reviews often reflects conceptual alignment rather than shared empirical foundations [48].

Analysing the characteristics of transition programmes, the most reported one is the one-on-one preceptorship, where experienced nurses serve as clinical guides, socializers, and competency validators [34,36,39,42,43]. These programmes often incorporate supernumerary practice time, allowing new graduates to immerse themselves in clinical duties without the immediate pressure of an independent patient assignment [35,36]. Educational strategies move beyond traditional lectures to include simulation-based learning, which provides a safe environment to practice high-stakes, low-frequency clinical scenarios. To foster clinical reasoning, programmes utilise active learning methods such as small group case studies, clinical conferences, and staggered didactic education that distributes content across the first year to align with the learner’s evolving needs [33,37,39,40,43,44]. Furthermore, reflective practice through journaling and group debriefing sessions provides essential psychosocial support, helping novices navigate the stages of “doing, being, and knowing” while mitigating the effects of transition shock [34,39,40,41]. This comprehensive approach ensures that new hires are not left to acclimate in isolation, ultimately bolstering their clinical competence and professional confidence [34,36,40,41,43]. The programmes have different durations, ranging from a few days to 12 months. The longer duration is justified by the intention to reflect the learning curve of new hires [24,34,38,39,41,43].

Outcome heterogeneity further compounds these challenges. Across reviews, outcomes are variably defined, operationalised, and measured, with inconsistent use of validated instruments, differing follow-up periods, and limited attention to causal attribution [24,25,35,36,37,38,39,40,41,42,43,44,45,46]. This variability is particularly pronounced for organisational and system-level outcomes, such as retention, turnover, and workforce stability, which are frequently reported but rarely examined using comparable metrics or longitudinal designs. As a result, synthesis at the review level often relies on narrative aggregation rather than quantitative consolidation, reinforcing descriptive rather than explanatory conclusions.

Across the included reviews, outcomes associated with nurse transition programmes are reported at professional, organisational, and, more marginally, patient or clinical levels. However, the interpretation of these outcomes is constrained by substantial heterogeneity in outcome definitions, measurement approaches, and analytical framing, limiting the extent to which findings can be considered cumulative or directly comparable across reviews [24,25,35,36,37,38,39,40,41,42,43,44,45,46].

At the professional level, outcomes related to clinical competence, confidence, and perceived readiness for practice are the most consistently reported and the most uniformly positive [49]. Reviews focusing on orientation, transition-to-practice, and residency models frequently describe improvements in newly graduated or newly qualified nurses’ clinical skills, self-confidence, and adaptation to professional roles. These outcomes are particularly prominent in earlier reviews and in integrative and systematic reviews of primary studies, where individual-level change constitutes the primary analytic focus. Nevertheless, even within this domain, outcome measurement is highly variable, often relying on self-reported indicators or heterogeneous assessment tools, which limits interpretive precision and cross-review comparability.

Professional socialisation, job satisfaction, and sense of belonging are also reported as favourable outcomes in several reviews, although findings in this domain are more mixed. While some reviews describe enhanced professional integration and improved work-related attitudes, others report inconsistent or context-dependent effects [24,37,43]. These variations appear closely linked to programme characteristics, such as the quality of preceptorship relationships, organisational support, and workload conditions, underscoring the difficulty of attributing professional socialisation outcomes solely to programme participation. Moreover, stress reduction and improved transition experiences are reported less consistently, with several reviews highlighting persistent stress and role overload despite programme implementation, suggesting that transition interventions may mitigate but not eliminate the challenges associated with early professional practice.

Organisational outcomes, particularly retention, turnover, and intention to stay, are frequently cited across reviews, especially those examining nurse residency and transition-to-practice programmes [49,50,51]. Most reviews report positive associations between structured transition programmes and retention-related outcomes; however, these findings are characterised by important methodological limitations. Retention is inconsistently defined and operationalised, follow-up periods vary widely, and causal inferences are rarely supported by robust comparative designs. As a result, while the direction of association is generally favourable, the strength and durability of organisational effects remain uncertain. More recent umbrella reviews place greater emphasis on these organisational outcomes, reflecting a shift in analytical priorities rather than a consolidation of stronger evidence.

Evidence related to broader service-level or workforce outcomes, such as staffing stability, workforce sustainability, or organisational performance, is limited and fragmented [24,25,35,36,37,38,39,40,41,42,43,44,45,46]. Where such outcomes are addressed, findings are typically descriptive and derived from secondary synthesis rather than direct empirical evaluation. Economic outcomes, including cost-effectiveness or return on investment, are rarely examined and, when reported, are based on indirect or proxy indicators. This scarcity of economic and system-level evidence constrains the capacity of reviews to inform organisational decision-making beyond general claims of potential benefit.

Patient or clinical outcomes represent the least developed outcome domain across the review literature. Most reviews either do not report patient-level outcomes or refer to them indirectly, often citing patient safety or quality of care as assumed benefits rather than empirically assessed endpoints. When patient outcomes are mentioned, the evidence is typically inconclusive or based on surrogate measures, reflecting the secondary status of patient-level effects within the conceptualisation of nurse transition programmes. This pattern suggests that, despite frequent rhetorical linkage between nurse transition support and patient care quality, patient outcomes remain peripheral within review-level syntheses.

Overall, the outcome evidence synthesised across reviews reflects a clear imbalance between individual-level and organisational or system-level domains. Positive professional outcomes are more consistently reported, whereas organisational outcomes are less robustly supported, and patient-level outcomes remain largely unexamined. Consequently, the existing review literature supports cautious optimism about the professional benefits of nurse transition programmes, while offering more tentative, methodologically constrained conclusions about their organisational and system-level impact.

### 4.1. Explaining Conceptual Plurality in Nurse Transition Programmes

As used in this review, conceptual plurality refers not only to the multiplicity of terms used in the literature, but also to substantive variation in how nurse transition programmes are defined, structured, and embedded within organisations. The conceptual plurality observed across the review literature reflects several structural factors shaping the development of nurse transition programmes rather than merely inconsistent terminology. It partly derives from the historical evolution of transition support models, which have expanded over time from locally developed orientation initiatives to more structured programmes integrating educational, professional, and psychosocial components [24,25,35,36,37,38,39,40,41,42,43,44,45,46]. Differences between healthcare systems and national workforce policies additionally influence how transition programmes are conceptualised and implemented, generating variation in programme structure and terminology across contexts. In this regard, many transition models incorporate concepts originating in distinct educational and professional development traditions, such as mentorship, preceptorship, residency, and organisational onboarding, thereby contributing to overlapping conceptual frameworks. In addition, organisational adaptation to local workforce needs and resource constraints further diversifies programme design, resulting in programmes with similar labels but substantially different structures and implementation strategies. These elements suggest that conceptual plurality reflects the historically, institutionally, and epistemologically diverse processes underlying nurse transition support, highlighting the need for greater conceptual clarification to strengthen comparability and cumulative evidence development.

### 4.2. Implications for Future Research

The findings of this umbrella review identify several priorities for future research to strengthen the evidence base on nurse transition programmes and enhance their cumulative value. First, there is a clear need for greater conceptual clarity and explicit theorisation of nurse transition interventions. Future primary studies and reviews should move beyond nominal labelling and explicitly describe programme characteristics, including duration, level of formalisation, core components, and organisational embedding. The adoption of shared conceptual frameworks or taxonomies would support greater comparability across studies and facilitate more meaningful synthesis at both primary and review levels.

Second, future reviews should explicitly justify the aggregation of interventions under common labels and clearly articulate assumptions regarding programme equivalence. Where substantial heterogeneity exists, subgroup analyses or typology-based syntheses may be more appropriate than broad narrative aggregation. This is particularly relevant for reviews addressing onboarding, transition-to-practice, and residency models, which are frequently conflated despite substantive differences in scope and intent.

Third, greater methodological attention is needed in the measurement and reporting of outcomes, especially at organisational and system levels. Future research should prioritise the use of standardised, validated outcome measures and adopt longitudinal designs capable of capturing sustained effects on retention, turnover, workforce stability, and organisational performance. The limited and inconsistent evidence currently available on economic outcomes highlights the need for robust evaluations of cost-effectiveness and return on investment, particularly given the resource-intensive nature of many transition programmes.

Fourth, patient-level outcomes remain largely unexplored within the review literature and should be addressed more explicitly in future primary studies and syntheses. While nurse transition programmes are frequently justified in terms of patient safety and quality of care, empirical examination of these outcomes is rare. Future evaluations should more explicitly assess patient safety and quality-of-care outcomes, so that nurse transition programmes are not evaluated solely in terms of nurse satisfaction, professional adjustment, or retention, but also in relation to their potential contribution to safer patient care.

### 4.3. Implications for Nursing Management

From a nursing management perspective, the conceptual plurality identified across reviews should not obscure the presence of recurring programme features that can guide implementation. Although no single transition model emerged as universally superior, the review suggests that effective programmes commonly include a structured entry pathway, protected time for learning, consistent support from trained preceptors or mentors, opportunities for supervised practice and formative feedback, and clear organisational commitment in terms of coordination and resources. Nursing directors may therefore use these recurring components as a pragmatic framework when designing or refining local transition programmes, while adapting programme duration, intensity, and delivery to workforce needs and clinical context.

### 4.4. Limitations

This umbrella review has several limitations that should be considered when interpreting its findings. First, the review is inherently dependent on the scope, quality, and reporting practices of the included reviews. Although methodological quality was assessed using the JBI Appraisal Checklist, variability in review rigour, transparency, and synthesis approaches inevitably influenced the strength and interpretability of the findings.

Second, the decision to include multiple forms of evidence synthesis was methodologically justified to capture the full breadth of the review-level literature. However, this inclusivity also introduced substantial heterogeneity, limiting the extent to which findings could be synthesised quantitatively and necessitating reliance on narrative and thematic approaches.

Third, the decision to include reviews of reviews alongside reviews of primary studies represents a deliberate but non-standard methodological choice, motivated by the study’s focus on conceptual plurality in the review-level evidence base. We acknowledge that this design introduces a theoretical risk of double-counting of primary studies. To quantify this risk, a sensitivity analysis was performed by recalculating the CCA over the expanded pool of 14 reviews, incorporating the primary studies transitively contributed by the two umbrella/meta-reviews; the recalculated value remained below the 5% threshold conventionally associated with slight overlap. Nevertheless, the residual risk of informational overlap cannot be entirely excluded, and this methodological choice should be weighed when interpreting the findings.

Fourth, although the overlap among primary studies was formally assessed using the CCA, the analysis was constrained by incomplete reporting of primary study inclusion criteria in some reviews. As a result, overlap estimates should be interpreted as indicative rather than definitive. Nevertheless, the consistently low CCA supports the conclusion of limited communality across reviews.

Fifth, this review was restricted to publications in English, which may have resulted in the exclusion of relevant reviews published in other languages. In addition, grey literature was not systematically searched, potentially omitting non-peer-reviewed syntheses or organisational reports that may address implementation aspects of nurse transition programmes.

Finally, the umbrella review focused on review-level evidence and did not reanalyse primary studies. Consequently, conclusions are necessarily constrained by the analytical frames and outcome selections adopted in the included reviews. This limitation is intrinsic to the design of umbrella reviews but reinforces the importance of interpreting findings in light of the conceptual and methodological characteristics of the secondary evidence base.

## 5. Conclusions

This umbrella review demonstrates that review-level evidence on nurse transition programmes is marked by pronounced conceptual plurality, methodological heterogeneity, and limited overlap of underlying primary studies, resulting in a fragmented evidence base in which similarly labelled programmes often represent substantively different interventions grounded in largely distinct empirical foundations. Consequently, apparent convergence across reviews reflects shared terminology and shifting analytical priorities rather than cumulative confirmation of programme effectiveness. Within this heterogeneous landscape, several recurring components emerge across the analysed induction programmes; most notably, preceptorship, competency validation, and active learning methodologies, such as simulations, debriefings, and case studies, constitute the predominant interventions. Regarding the outcomes associated with such programmes, the findings support cautious confidence in the professional benefits of nurse transition programmes, particularly in relation to competence, confidence, and early professional adjustment. However, evidence for organisational and system-level outcomes, including retention, workforce stability, and cost-effectiveness, remains inconsistent and methodologically constrained, while patient-level outcomes are largely absent from review-level syntheses. The state-of-the-art described by this review provides a critical foundation for future research by clarifying the conceptual, thematic, and evidentiary structure of the available literature regarding nurse transition interventions. Future studies should embed these findings within an operational framework to evaluate their impact on professional and organisational contexts.

## Figures and Tables

**Table 1:** Conceptual framework.

Component	Key Terms
Population	Newly hired nurses (newly, nurs*)
Intervention	Onboarding, orientation, transition programmes, mentorship, preceptorship
Comparator	Any or no comparator
Outcomes	Any professional or organisational outcomes

**Table 2:** Final search strategy and information sources.

Database	Search Strings	Records Retrieved	Date of Search
PubMed	(“orientation*”[tiab] OR “onboarding*”[tiab] OR “transition* program*”[tiab] OR “mentorship*”[tiab] OR “preceptorship”[tiab]) AND (“nurs*”[tiab] AND “newly”[tiab])	496	10 January 2026
CINAHL	((TI orientation* OR AB orientation*) OR (TI onboarding* OR AB onboarding*) OR (TI “transition* program*” OR AB “transition* program*”) OR (TI mentorship* OR AB mentorship*) OR (TI preceptorship OR AB preceptorship)) AND ((TI nurs* OR AB nurs*) AND (TI newly OR AB newly))	539	10 January 2026
Scopus	(TITLE-ABS(orientation*) OR TITLE-ABS(onboarding*) OR TITLE-ABS(“transition* program*”) OR TITLE-ABS(mentorship*) OR TITLE-ABS(preceptorship)) AND (TITLE-ABS(nurs*) AND TITLE-ABS(newly))	537	10 January 2026
Web of Science	((TI = orientation* OR AB = orientation*) OR (TI = onboarding* OR AB = onboarding*) OR (TI = “transition* program*” OR AB = “transition* program*”) OR (TI = mentorship* OR AB = mentorship*) OR (TI = preceptorship OR AB = preceptorship)) AND ((TI = nurs* OR AB = nurs*) AND (TI = newly OR AB = newly))	339	10 January 2026

**Table 3:** Characteristics of the included reviews.

Review	Type of Review	Study Designs Included	Critical Appraisal	Synthesis Approach
Park et al., 2010 [41]	Integrative review	Quantitative	Partial	Narrative synthesis
Edwards et al., 2015 [38]	Systematic review	Quantitative and qualitative	Yes (JBI)	Narrative synthesis
Pertiwi and Hariyati, 2019 [42]	Systematic review	Quantitative and qualitative	Yes (JBI)	Narrative synthesis
Ernawaty et al., 2024 [39]	Scoping review	Quantitative and qualitative	Not applicable	Descriptive mapping
Edward et al., 2017 [37]	Integrative review	Mixed	Partial	Narrative synthesis
Rush et al., 2019 [45]	Integrative review	Mixed	Partial	Narrative synthesis
Quek and Shorey, 2018 [43]	Integrative review	Quantitative and qualitative	Yes (JBI)	Thematic synthesis
Zhang et al., 2016 [46]	Systematic review	Quantitative and qualitative	Partial	Narrative synthesis
Eckerson, 2018 [36]	Systematic review (doctoral thesis)	Quantitative	Yes	Narrative synthesis
Lima and Alzyood, 2024 [40]	Integrative review	Quantitative and qualitative	Partial	Narrative synthesis
Reebals et al., 2021 [44]	Integrative review	Mixed	Partial	Narrative synthesis
Kenny et al., 2021 [24]	Mixed-methods systematic review	Quantitative, qualitative, mixed	Yes (MMAT)	Narrative + thematic synthesis
Berthelsen et al., 2025 [25]	Umbrella review	Systematic reviews	Yes	Review-of-reviews synthesis
Reinhard et al., 2017 [35]	Umbrella/meta-review	Systematic reviews	Yes (AMSTAR)	Review-of-reviews synthesis

**Table 4:** Key characteristics of included reviews, including aims, evidence base, populations, settings, and outcomes.

Review	Aim of the Review	Review Period	Number of Primary Studies/ Evidence Base	Target Population/Healthcare Setting	Main Focus and Intervention	Key Outcomes Assessed
Park et al., 2010 [41]	To examine the effectiveness of orientation and transition programmes for newly graduated nurses	1990–2007	17 primary studies	Newly graduated nurses/Acute care hospitals	Internship, residency, structured orientation, preceptorship, didactic classes	Retention, clinical competence, confidence
Edwards et al., 2015 [38]	To evaluate the effectiveness of strategies supporting the transition from student to qualified nurse	2000–2011	30 primary studies	Newly qualified nurses/Multiple clinical settings	Internship, residency, mentorship, preceptorship, simulation	Retention, clinical competence, job satisfaction, stress/anxiety reduction
Zhang et al., 2016 [46]	To evaluate the effectiveness of mentoring programmes for newly graduated nurses	Up to 2014	9 primary studies	Newly graduated nurses/Hospital	Formal/informal mentorship, mentor selection/training, matching	Turnover reduction, clinical competence, confidence, stress reduction, cost-effectiveness
Edward et al., 2017 [37]	To examine transition-to-practice support strategies and outcomes	1980–2017	15 primary studies	Newly qualified nurses/Multiple clinical settings	Nurse residency programme, preceptorship, mentorship, simulation, distance learning	Retention, turnover reduction, clinical competence, confidence, job satisfaction, stress reduction
Reinhard et al., 2017 [35]	To synthesise evidence on nurse residency and transition programmes	2000–2015	177 studies from 8 systematic reviews	Newly graduated nurses/Hospital	Nurse residency programme, preceptorship, mentorship, simulation, distance learning	Retention, turnover reduction, clinical competence, confidence, job satisfaction, stress reduction
Eckerson, 2018 [36]	To evaluate nurse residency programmes	Up to 2017	12 primary studies	Newly graduated nurses/Hospital	Nurse residency programme, preceptor mentorship, simulation, didactic case studies, peer support	Retention, job satisfaction, cost-effectiveness (ROI)
Quek and Shorey, 2018 [43]	To explore experiences and perceptions of preceptorship	2006–2016	20 primary studies	Newly graduated nurses, preceptors/Hospital	Preceptorship, preceptor education, socialisation, psychosocial support	Retention, clinical competence, confidence, job satisfaction, stress reduction
Pertiwi and Hariyati, 2019 [42]	To identify effective hospital orientation programmes for newly graduated nurses	2008–2018	14 primary studies	Newly graduated nurses/Hospital	Preceptorship, mentorship, didactic classes, simulation	Retention, turnover reduction, clinical competence, job satisfaction, stress reduction
Rush et al., 2019 [45]	To identify best practices of formal transition programmes	2000–2018	76 primary studies	Newly graduated nurses/Acute care	Nurse residency programme, extended preceptorship, mentorship, staggered education, peer support	Retention, turnover reduction, clinical competence, cost-effectiveness
Kenny et al., 2021 [24]	To document transition interventions and associated outcomes	1990–2020	130 primary studies	Graduate nurses/Multiple healthcare settings	Residency, mentorship, preceptorship, orientation tracks, graduate retreats, peer support	Retention, clinical competence, confidence, job satisfaction
Reebals et al., 2021 [44]	To identify barriers and facilitators to transition to practice	2014–2020	23 primary studies	Newly graduated nurses/Hospital	Structured transition programmes, residency/fellowship, mentorship, preceptor training, stress management	Retention, turnover reduction, clinical competence, stress reduction
Ernawaty et al., 2024 [39]	To map components, duration, and outcomes of orientation programmes	2018–2023	14 primary studies	New nurses/Hospital	Preceptorship, mentorship, clinical rotation, simulation, peer support, reflection seminars	Turnover reduction, clinical competence, confidence, job satisfaction, stress reduction
Lima and Alzyood, 2024 [40]	To assess the impact of preceptorship in critical care settings	2010–2022	9 primary studies	Newly qualified nurses, preceptors/Critical care	Preceptorship, preceptor training, supernumerary time, protected time	Clinical competence, confidence, stress reduction, work readiness, socialisation/belonging
Berthelsen et al., 2025 [25]	To synthesise evidence on introduction programmes for nurses	Up to 2025	84 studies (70 unique) from 5 systematic reviews	Newly hired nurses/Multiple healthcare settings	Preceptorship, mentorship, residency, internship, externship, preceptor training	Retention, turnover reduction, engagement, clinical competence, confidence

**Table 5:** Transition Programme models and defining features.

Programme Type	Core Component	Typical Duration	Level of Formalisation	Target Population	Reviews Reporting This Type
Orientation programmes	Classroom education; unit orientation; supervised practice; feedback, simulation	Short-term to medium	Variable	Newly graduated nurses	[38,39,41,42]
Transition-to-practice programmes	Structured education; supervised clinical practice; professional socialisation; competency development, simulation	Medium to extended	Semi-formal to formal	Newly graduated nurses	[24,44,45]
Nurse residency programmes	Classroom education; extended supervised practice; competency-based progression; evaluation, simulation	Extended (6–12 months)	Formal	Newly hired nurses (mostly newly graduated nurses)	[25,35,36,45]
Preceptorship-based programmes	One-to-one preceptorship; feedback; experiential learning, simulation	Variable	Variable	Newly graduated nurses (sometimes mixed)	[37,40,43]
Mentorship-based programmes	Mentoring relationships; psychosocial support; professional development, simulation	Variable	Semi-formal	Newly graduated nurses	[35,43]
Hybrid programmes	Orientation + residency/transition + mentorship, simulation	Variable	Variable	Newly graduated nurses	[24,38]

**Table 6:** Facilitators and barriers to the implementation of transition programmes.

Level	Facilitators	Barriers	Reviews Reporting
Individual	Motivation and readiness of new nurses; engagement; proactive learning behaviours	Stress, anxiety, lack of confidence, and role overload	[24,38,41]
Team/interpersonal	Consistent and trained preceptors; positive preceptor–preceptee relationships; peer support; team acceptance	Inconsistent preceptorship; lack of time for supervision; preceptor burnout	[37,40,43]
Organisational	Leadership commitment; structured programmes; protected learning time; programme coordinators; resource allocation	Staffing shortages; workload pressure; insufficient resources; variability in programme implementation	[25,44,45]
System/structural	Alignment with workforce strategies; policy support for transition programmes	Financial constraints; lack of standardisation; limited sustainability and cost-effectiveness data	[35,38]

**Table 7:** Professional and organisational outcomes associated with transition programmes across included reviews.

Outcome Domain	Outcomes Reported	Direction of Association	Reviews Reporting
Professional—competence and confidence	Increased clinical competence; improved confidence and work readiness	Mostly positive	[38,39,41,42,45]
Professional—socialisation and satisfaction	Improved professional socialisation; higher job satisfaction; sense of belonging	Positive to mixed	[24,37,43]
Professional—stress and transition experience	Reduced transition-related stress; improved coping	Mixed	[44]
Organisational—retention and turnover	Higher retention; reduced turnover; improved intent to stay	Positive (inconsistently measured)	[25,35,36,38,45]
Organisational—service-level outcomes	Limited evidence on staffing stability and workforce outcomes	Mixed/limited	[24,25]
Patient/clinical outcomes	Rarely reported; indirect or proxy measures only	Inconclusive	[24,25,35,36,37,38,39,40,41,42,43,44,45,46]

## Data Availability

The data supporting the findings of this umbrella review are based on previously published reviews and are reported in the main text, tables, and figures. No new primary data were generated. Derived datasets used for evidence mapping and thematic synthesis are available from the corresponding author upon reasonable request.

## Public Involvement Statement

No public involvement in any aspect of this research.

## Guidelines and Standards Statement

This manuscript was drafted against the Preferred Reporting Items for Systematic reviews and Meta-Analyses (PRISMA) statement 2020.

## Abbreviations

The following abbreviations are used in this manuscript:

CCA	Corrected Covered Area
PRISMA	Preferred Reporting Items for Systematic Reviews and Meta-Analyses
JBI	Joanna Briggs Institute

## References

[1] Amini Rarani S. (2026). Nursing Workforce in Collapse: A Narrative Review of Global Shortages, Burnout, and the Future of Health System Resilience. Int. J. Afr. Nurs. Sci..

[2] Bourgault A.M. (2022). The Nursing Shortage and Work Expectations Are in Critical Condition: Is Anyone Listening?. Crit. Care Nurse.

[3] Magon A., Caruso R. (2023). Addressing a Potential Crisis in the Italian National Health System. Lancet.

[4] Vázquez-Calatayud M., Eseverri-Azcoiti M.C. (2023). Retention of Newly Graduated Registered Nurses in the Hospital Setting: A Systematic Review. J. Clin. Nurs..

[5] Narbona-Gálvez Á., García-Iglesias J.J., Ayuso-Murillo D., Fontán-Vinagre G., Gómez-Salgado J., Allande-Cussó R., Fagundo-Rivera J., Macías-Toronjo I., Ruiz-Frutos C. (2024). Stress in Novice Nurses in New Work Environments: A Systematic Review. Front. Public Health.

[6] Wakefield E., Innes K., Dix S., Brand G. (2023). Belonging in High Acuity Settings: What Is Needed for Newly Graduated Registered Nurses to Successfully Transition? A Qualitative Systematic Review. Nurse Educ. Today.

[7] McClain A.R., Palokas M., Christian R., Arnold A. (2022). Retention Strategies and Barriers for Millennial Nurses: A Scoping Review. JBI Evid. Synth..

[8] Williamson L., Burog W., Taylor R.M. (2022). A Scoping Review of Strategies Used to Recruit and Retain Nurses in the Health Care Workforce. J. Nurs. Manag..

[9] Penconek T., Tate K., Lartey S.A., Polat D., Bernardes A., Moreno Dias B., Nuspl M., Cummings G.G. (2024). Factors Influencing Nurse Manager Retention, Intent to Stay or Leave and Turnover: A Systematic Review Update. J. Adv. Nurs..

[10] Conroy N., Patton D., Moore Z., O’Connor T., Nugent L., Derwin R. (2023). The Relationship between Transformational Leadership and Staff Nurse Retention in Hospital Settings: A Systematic Review. J. Nurs. Manag..

[11] Chang H.E., Cho S. (2023). Turnover Intention and Retention of Newly Licensed Nurses in Their First Job: A Longitudinal Study. Int. Nurs. Rev..

[12] McIntyre N., Crilly J., Elder E. (2024). Factors That Contribute to Turnover and Retention amongst Emergency Department Nurses: A Scoping Review. Int. Emerg. Nurs..

[13] Woodward K.F., Willgerodt M. (2022). A Systematic Review of Registered Nurse Turnover and Retention in the United States. Nurs. Outlook.

[14] Lay K.S.M., Masingboon K. (2025). Turnover Prevalence and the Relationship between Transition Shock and Turnover Intention among New Nurses: A Meta-Analysis. Int. J. Nurs. Stud. Adv..

[15] Södergård E., Juntunen J., Kuivila H., Tomietto M., Mikkonen K. (2025). THE Effect of Mentoring Programmes on Newly Graduated Nurses’ Retention and Turnover: An Umbrella Review. J. Adv. Nurs..

[16] Joseph B., Jacob S., Lam L., Rahman M.A. (2022). Factors Influencing the Transition and Retention of Mental Health Nurses during the Initial Years of Practice: Scoping Review. J. Nurs. Manag..

[17] Masso M., Sim J., Halcomb E., Thompson C. (2022). Practice Readiness of New Graduate Nurses and Factors Influencing Practice Readiness: A Scoping Review of Reviews. Int. J. Nurs. Stud..

[18] Baharum H., Ismail A., McKenna L., Mohamed Z., Ibrahim R., Hassan N.H. (2023). Success Factors in Adaptation of Newly Graduated Nurses: A Scoping Review. BMC Nurs..

[19] Li Y., Wang C., Tan W., Jiang Y. (2023). The Transition to Advanced Practice Nursing: A Systematic Review of Qualitative Studies. Int. J. Nurs. Stud..

[20] See E.C.W., Koh S.S.L., Baladram S., Shorey S. (2023). Role Transition of Newly Graduated Nurses from Nursing Students to Registered Nurses: A Qualitative Systematic Review. Nurse Educ. Today.

[21] LeJeune K. (2023). Enhancing Nurse Leadership Engagement Through Formalized Orientation Programs: An Integrative Review. JONA J. Nurs. Adm..

[22] Kurnat-Thoma E., Ganger M., Peterson K., Channell L. (2017). Reducing Annual Hospital and Registered Nurse Staff Turnover—A 10-Element Onboarding Program Intervention. Sage Open Nurs..

[23] Torres D.A., Jeske L., Marzinski S.J., Oleson R., Hook M.L. (2022). Best Fit Orientation: An Innovative Strategy to Onboard Newly Licensed Nurses. J. Nurses Prof. Dev..

[24] Kenny A., Dickson-Swift V., McKenna L., Charette M., Rush K.L., Stacey G., Darvill A., Leigh J., Burton R., Phillips C. (2021). Interventions to Support Graduate Nurse Transition to Practice and Associated Outcomes: A Systematic Review. Nurse Educ. Today.

[25] Berthelsen C., Hansen C.A. (2025). Content and Effect of Introduction Programmes to Increase Retention and Decrease Turnover of Newly Graduated Nurses in Hospitals: Umbrella Review. J. Clin. Nurs..

[26] Santos W.M.D., Secoli S.R., Püschel V.A.d.A. (2018). The Joanna Briggs Institute Approach for Systematic Reviews. Rev. Lat. Am. Enferm..

[27] Brignardello-Petersen R., Santesso N., Guyatt G.H. (2025). Systematic Reviews of the Literature: An Introduction to Current Methods. Am. J. Epidemiol..

[28] Torre M., Arrigoni C., Caruso R., Magon A. (2026). Onboarding Programs for Newly Hired Nurses in Healthcare Organizations: A Protocol for a Systematic Review of Systematic Reviews. Figureshare.

[29] Page M.J., McKenzie J.E., Bossuyt P.M., Boutron I., Hoffmann T.C., Mulrow C.D., Shamseer L., Tetzlaff J.M., Akl E.A., Brennan S.E. (2021). The PRISMA 2020 Statement: An Updated Guideline for Reporting Systematic Reviews. Int. J. Surg..

[30] Zhou C., Fabiano N., Gupta A., Wong S., Cobey K.D., Moher D., Ebrahimzadeh S., Ng J.Y., Dragioti E., Shin J.I. (2026). Guidance for Umbrella Reviews of Observational Studies: A Scoping Review. JCPP Adv..

[31] Bougioukas K.I., Vounzoulaki E., Mantsiou C.D., Savvides E.D., Karakosta C., Diakonidis T., Tsapas A., Haidich A.-B. (2021). Methods for Depicting Overlap in Overviews of Systematic Reviews: An Introduction to Static Tabular and Graphical Displays. J. Clin. Epidemiol..

[32] Schiavenato M., Chu F. (2021). PICO: What It Is and What It Is Not. Nurse Educ. Pract..

[33] Hilton M. (2024). JBI Critical Appraisal Checklist for Systematic Reviews and Research Syntheses. J. Can. Health Libr. Assoc..

[34] Kirvalidze M., Abbadi A., Dahlberg L., Sacco L.B., Calderón-Larrañaga A., Morin L. (2023). Estimating Pairwise Overlap in Umbrella Reviews: Considerations for Using the Corrected Covered Area (CCA) Index Methodology. Res. Synth. Methods.

[35] Reinhard P. (2017). New Graduate Nurse Transition Programs: A Meta-Review. Doctoral Dissertation.

[36] Eckerson C.M. (2018). The Impact of Nurse Residency Programs in the United States on Improving Retention and Satisfaction of New Nurse Hires: An Evidence-Based Literature Review. Nurse Educ. Today.

[37] Edward K., Ousey K., Playle J., Giandinoto J.-A. (2017). Are New Nurses Work Ready—The Impact of Preceptorship. An Integrative Systematic Review. J. Prof. Nurs..

[38] Edwards D., Hawker C., Carrier J., Rees C. (2015). A Systematic Review of the Effectiveness of Strategies and Interventions to Improve the Transition from Student to Newly Qualified Nurse. Int. J. Nurs. Stud..

[39] Ernawaty E., Hariati S., Saleh A. (2024). Program Components, Impact, and Duration of Implementing a New Nurse Orientation Program in Hospital Contexts: A Scoping Review. Int. J. Nurs. Stud. Adv..

[40] Lima M.S., Alzyood M. (2024). The Impact of Preceptorship on the Newly Qualified Nurse and Preceptors Working in a Critical Care Environment: An Integrative Literature Review. Nurs. Crit. Care.

[41] Park M., Jones C.B. (2010). A Retention Strategy for Newly Graduated Nurses: An Integrative Review of Orientation Programs. J. Nurses Staff Dev. (JNSD).

[42] Pertiwi R.I., Hariyati R.T.S. (2019). Effective Orientation Programs for New Graduate Nurses: A Systematic Review. Enfermería Clínica.

[43] Quek G.J.H., Shorey S. (2018). Perceptions, Experiences, and Needs of Nursing Preceptors and Their Preceptees on Preceptorship: An Integrative Review. J. Prof. Nurs..

[44] Reebals C., Wood T., Markaki A. (2022). Transition to Practice for New Nurse Graduates: Barriers and Mitigating Strategies. West. J. Nurs. Res..

[45] Rush K.L., Janke R., Duchscher J.E., Phillips R., Kaur S. (2019). Best Practices of Formal New Graduate Transition Programs: An Integrative Review. Int. J. Nurs. Stud..

[46] Zhang Y., Qian Y., Wu J., Wen F., Zhang Y. (2016). The Effectiveness and Implementation of Mentoring Program for Newly Graduated Nurses: A Systematic Review. Nurse Educ. Today.

[47] Morgan P., Barnes H., Batchelder H.R., Tuttle B., Covelli A.F., Everett C., Jackson G.L., Anglin L., Pate N.O., Dieter P. (2023). NP and PA Transition to Practice: A Scoping Review of Fellowships and Onboarding Programs. JAAPA.

[48] Ying X., Bougioukas K.I., Pieper D., Mayo-Wilson E. (2025). Weighted Corrected Covered Area (wCCA): A Measure of Informational Overlap among Reviews. Res. synth. Methods.

[49] Wynne K., Mwangi F., Onifade O., Abimbola O., Jones F., Burrows J., Lynagh M., Majeed T., Sharma D., Bembridge E. (2024). Readiness for Professional Practice among Health Professions Education Graduates: A Systematic Review. Front. Med..

[50] Galanis P., Moisoglou I., Papathanasiou I.V., Malliarou M., Katsiroumpa A., Vraka I., Siskou O., Konstantakopoulou O., Kaitelidou D. (2024). Association between Organizational Support and Turnover Intention in Nurses: A Systematic Review and Meta-Analysis. Healthcare.

[51] Thin S.M., Chongmelaxme B., Watcharadamrongkun S., Kanjanarach T., Sorofman B.A., Kittisopee T. (2022). A Systematic Review on Pharmacists’ Turnover and Turnover Intention. Res. Soc. Adm. Pharm..

